# The subsets of blood circulating T-cells associated with the development and prognosis of coinfection in patients with critical COVID-19

**DOI:** 10.3389/fimmu.2025.1586302

**Published:** 2025-05-09

**Authors:** Xingming Li, Hongqiong Peng, Yunchuan Wang, Shiying He, Xueting Yang, Jiayue Chen

**Affiliations:** ^1^ Department of Emergency, Sichuan Academy of Medical Sciences, Sichuan Provincial People’s Hospital, Chengdu, China; ^2^ Department of Pathology, Sichuan Academy of Medical Sciences, Sichuan Provincial People’s Hospital, Chengdu, China

**Keywords:** COVID-19, critical pneumonia, bacterial coinfection, immunological characteristics, lymphocyte subsets

## Abstract

**Background:**

A secondary bacterial infection, which has a high incidence in patients with critical coronavirus disease 2019 (COVID-19), has been proven to have an association with increased mortality. Adaptive immune responses have been detected in almost all COVID-19 cases. This study aimed to determine whether the levels of immune-inflammatory factors are associated with coinfection in patients with critical COVID-19.

**Methods:**

Patients with a confirmed critical severe acute respiratory syndrome coronavirus 2 (SARS-CoV-2) infection were enrolled in this single-center cohort study. Clinical data and venous blood samples were collected on the day of hospital admission. All patients were divided into two groups according to the presence of bacterial coinfection or absence of bacterial coinfection, which were then divided into two groups (survived group and deceased group) based on the outcome of the disease during hospitalization.

**Results:**

Patients with coinfection had a higher mortality rate (83.3% VS 50.0%, P<0.001) and longer hospital stays (25.15 VS 13.80d, P<0.001). We observed that patients who developed coinfection tended to have a significantly lower number of CD4+ T cells (121.19 VS 207.83cells/µL, P=0.001) and CD8+ T cells (79 VS 158cells/µL, P=0.006) and a higher proportion of CD4+CD8+ double-positive T (DPT) cells (3.66% VS 1.91%, P=0.011) on the day of hospital admission. The tests for inflammatory cytokines showed a higher level of IL-4 (0.99 VS 0.42pg/mL, P<0.001) and IL-6 (109.60 VS 63.59pg/mL, P=0.009) in coinfection group. And the multivariant analyses also revealed that CD4+ cell counts < 199.5cells/µL, CD8+ cell counts < 124.5cells/µL, IL4 > 0.535pg/mL, IL6 > 388.9pg/mL could be independent risk factors for coinfection. Moreover, in the coinfection group, we observed that the deceased patients had a lower level of total lymphocytes, T cells, and albumin.

**Conclusion:**

Our study found that lymphocyte subsets and cytokines play an important role in predicting bacterial coinfection in patients with critical COVID-19. Lower levels of CD4+ and CD8+ cells and higher level of IL4 and IL6 in patients on the day of admission were significantly correlated with the development of coinfection the following days in the hospital.

## Introduction

Coronavirus disease 2019 (COVID-19), caused by the severe acute respiratory syndrome coronavirus 2 (SARS-CoV-2), has become a global public health challenge. According to the reports regarding patients from different districts in mainland China, while the signs and symptoms of most COVID-19 patients are usually mild to moderate, approximately 15–20% of individuals progress to severe interstitial pneumonia (1) with a 2–3% death rate (2). Secondary bacterial infection can be an important cause of mortality. A recent study reported that the prevalence of secondary bacterial infections was 18.4% (3). However, patients with critical COVID-19 are reported to have a 32.7% to 100% incidence of secondary bacterial infections (4). The presence of coinfection has been associated with increased mortality and prolonged hospital stay (5).

A previous study indicated that a higher white blood cell count, neutrophil count, and C-reactive protein (CRP) level were predictive of early bacterial coinfection in hospitalized patients with COVID-19 pneumonia (6). However, the role of cell-mediated immunity in association with COVID-19 coinfection has not been thoroughly elucidated. The adaptive immune system responds to pathogens in an antigen-specific manner, leading to protective immunity. T cell responses are detectable in nearly all patients infected with SARS-CoV-2 (7). Among the three major lymphocyte subsets of the adaptive immune system, the response of CD4+ T cells and B cells to SARS-CoV-2 is more prominent than that of CD8+ T cells (8) and are associated with the control of primary SARS-CoV-2 infection (9). Previous studies demonstrated that the level of CD8+ cells was an independent risk factor for the severity of COVID-19 (1). And it was reported that a specific phenotype of senescent effector CD8+ T cells exclusively present in critically ill patients with COVID-19 (10). Our study sought to examine whether levels of immune-inflammation factors and other clinical characteristics are associated with the development and prognosis of coinfection in critically ill patients with COVID-19.

## Methods

A total of 98 patients with polymerase chain reaction (PCR) confirmed SARS-CoV-2 infection in Sichuan Provincial People’s Hospital for COVID-19 infection were enrolled between December 2022 and January 2023. The inclusion criteria were as follows: (1) age > 18 years; (2) ICU admission due to respiratory failure, shock, or other organ failure; and (3) SARS-CoV-2 infection confirmed by PCR testing.

Patient clinical data, including age, sex, and medical history, were collected. Venous blood tests examining routine profiles, coagulation function, renal function, inflammatory cytokines, and lymphocyte subsets were performed on the day of hospital admission. Patients were divided into two groups according to the presence or absence of bacterial coinfection. Coinfection was defined by one or more positive microbiologicalevidence of bacterial coinfection (including positive hemoculture, sputum culture, urine culture, or other body fluid culture) obtained after confirmation of COVID-19 infection with the exclusion of the bacteria which considered to be colonization and contamination. Patients with bacterial coinfection were then divided into a survived group (n=9) and a deceased group (n=45) according to the outcome of the disease during hospitalization.

Statistical analyses were performed using the IBM SPSS Statistics version 25. The distribution normality of all continuous variables was assessed using the Shapiro–Wilk test. Variables with normal distribution were presented as means ± standard deviation and analyzed using the Student’s t-test, otherwise as medians (with interquartile ranges) and analyzed using the Mann–Whitney U test. Categorical variables are presented as percentages of the total and were analyzed using the chi-square test or Fisher’s exact test. A two-tailed P-value of < 0.05, was evaluated by receiver operating characteristic (ROC) curves and areas under the ROC curves (AUCs). For parameters that were significant based on univariate analyses, stepwise backward logistic regression was used to test the influence of the independent variables.

## Results

Among the 98 patients with COVID-19, 54 (55.1%) were diagnosed with confirmed bacterial infection, 30 of whom were infected by drug-resistant bacteria, including methicillin-resistant Staphylococcus aureus, carbapenem-resistant Acinetobacter baumannii, Klebsiella pneumoniae and pseudomonas aeruginosa. The identified seven bacteria were Acinetobacter baumannii, Klebsiella pneumoniae, Escherichia coli, Pseudomonas aeruginosa, Enterobacter aerogenes, Staphylococcus aureus and Enterococcus faecium. Coinfections were diagnosed approximately 11 ± 7 days after hospital admission (**Table 1**). There were no significant differences in age, sex, BMI (body mass index) and the proportion of participants with comorbidities of cancer or hematologic malignancy, diabetes, hypertension, chronic cardiovascular disease, autoimmune disease, chronic kidney disease, or chronic pulmonary disease between patients with and without coinfection. Patients with co-infection had higher mortality (83.3% VS 50.0%, P<0.001) and longer hospital stays (25.15 VS 13.80, P<0.001) during hospital admission. Regarding laboratory parameters, we did not find significant differences in routine blood examination and CRP or procalcitonin (PCT) levels between the two groups. The lymphocyte subset showed that patients with co-infection had a lower amount of CD4+ T cells (121.19 VS 207.83cells/µL, P=0.001) and CD8+ T cells (79 VS 158cells/µL, P=0.006) on the day of hospital admission, with a higher proportion of CD4+CD8+ double-positive T (DPT) (3.66% VS 1.91%, P=0.011). And the data of inflammatory cytokines showed a higher level of IL-4 (0.99 VS 0.42pg/mL, P<0.001) and IL-6 (109.60 VS 63.59pg/mL, P=0.009) in patients who develop with coinfection than those without.

**Table 1:** Clinical characteristics and laboratory indicators in patients with critical COVID-19.

Variable	Patients without coinfection	Patients with coinfection	*P-value*
Patients (n)	44	54	
Demographics			
Age (year, $\bar{x}\;\pm \;s$ )	73.75 ± 16.62	72.91 ± 15.45	0.463
Male [n (%)]	35 (79.5%)	46 (85.2%)	0.593
Hospital stay (days, $\bar{x}\;\pm \;s$ )	13.80 ± 9.92	25.15 ± 17.80	0.000
Mortality	22 (50.0%)	45 (83.3%)	0.000
Comorbidity			
Cancer [n (%)]	3 (6.8%)	2 (3.7%)	0.814
Diabetes mellitus [n (%)]	20 (45.5%)	17 (31.5%)	0.156
Hypertension	22 (50.0%)	30 (55.6%)	0.584
Chronic pulmonary disease	7 (15.9%)	11 (20.4%)	0.571
Chronic kidney disease	3 (6.8%)	7 (13.0%)	0.507
Laboratory parameters			
Leukocytes (*10^9^/L, $\bar{x}\;\pm \;s$ )	11.42 ± 4.97	11.20 ± 6.26	0.854
Neutrophils (*10^9^/L, $\bar{x}\;\pm \;s$ )	9.74 ± 4.40	9.93 ± 5.85	0.859
Lymphocytes (*10^9^/L, $\bar{x}\;\pm \;s$ )	0.70 ± 0.65	0.73 ± 0.58	0.771
Platelet (*10^9/L, $\bar{x}\;\pm \;s$ )	151.25 ± 96.20	171.15 ± 70.26	0.240
C-reactive protein (mmol/L, $\bar{x}\;\pm \;s$ )	96.75 ± 62.56	110.56 ± 74.24	0.332
Procalcitonin [ng/mL, *M* (*Q1*, *Q3*)]	0.75 (0.23,3.45)	0.58 (0.19,2.90)	0.385
Creatinine [µmol/L, *M* (*Q1*, *Q3*)]	125.50 (69.60,215.75)	101.65 (70.00,179.10)	0.500
ALB (g/L, $\bar{x}\;\pm \;s$ )	29.91 ± 5.14	30.64 ± 5.58	0.511
LDH (U/L, $\bar{x}\;\pm \;s$ )	541.34 ± 525.49	494.81 ± 239.38	0.562
BNP [pg/mL, *M* (*Q1*, *Q3*)]	248.05 (56.30,713.40)	115.25 (73.70,272.90)	0.382
T lymphocytes (% lymphocytes, $\bar{x}\;\pm \;s$ )	60.98 ± 15.62	59.94 ± 16.28	0.778
CD4+ [% lymphocytes, *M* (*Q1*, *Q3*)]	31.68 (25.36,42.78)	32.97 (23.78,40.75)	0.956
CD8+ (% lymphocytes, $\bar{x}\;\pm \;s$ )	26.72 ± 14.00	27.28 ± 12.60	0.852
CD4-CD8- [% lymphocytes, *M* (*Q1*, *Q3*)]	2.60 (1.27,3.50)	2.11 (1.09,3.64)	0.649
CD4+CD8+ (% lymphocytes, $\bar{x}\;\pm \;s$ )	1.91 ± 1.38	3.66 ± 4.08	0.011
T lymphocytes in blood (cells/µL, $\bar{x}\;\pm \;s$ )	280.61 ± 187.71	261.83 ± 166.19	0.657
CD4+ in blood (cells/µL, $\bar{x}\;\pm \;s$ )	207.83 ± 115.87	121.19 ± 76.19	0.001
CD8+ in blood [cells/µL, *M* (*Q1*, *Q3*)]	158 (88,197)	79 (43,134)	0.006
Ratio CD4+/CD8+ in blood ( $\bar{x}\;\pm \;s$ )	2.27 ± 2.18	1.74 ± 2.41	0.352
IL2 (pg/mL, $\bar{x}\;\pm \;s$ )	1.14 ± 0.84	1.45 ± 0.95	0.135
IL4 (pg/mL, $\bar{x}\;\pm \;s$ )	0.42 ± 0.31	0.99 ± 0.58	0.000
IL6 [pg/mL, *M* (*Q1*, *Q3*)]	63.59 (21.91,135.47)	109.60 (48.87,457.10)	0.009
IL10 [pg/mL, *M* (*Q1*, *Q3*)]	7.22 (4.10,12.06)	13.93 (4.52,49.72)	0.074
TNF-α (pg/mL, $\bar{x}\;\pm \;s$ )	0.50 ± 0.49	0.73 ± 0.90	0.196

**Figure 1:**
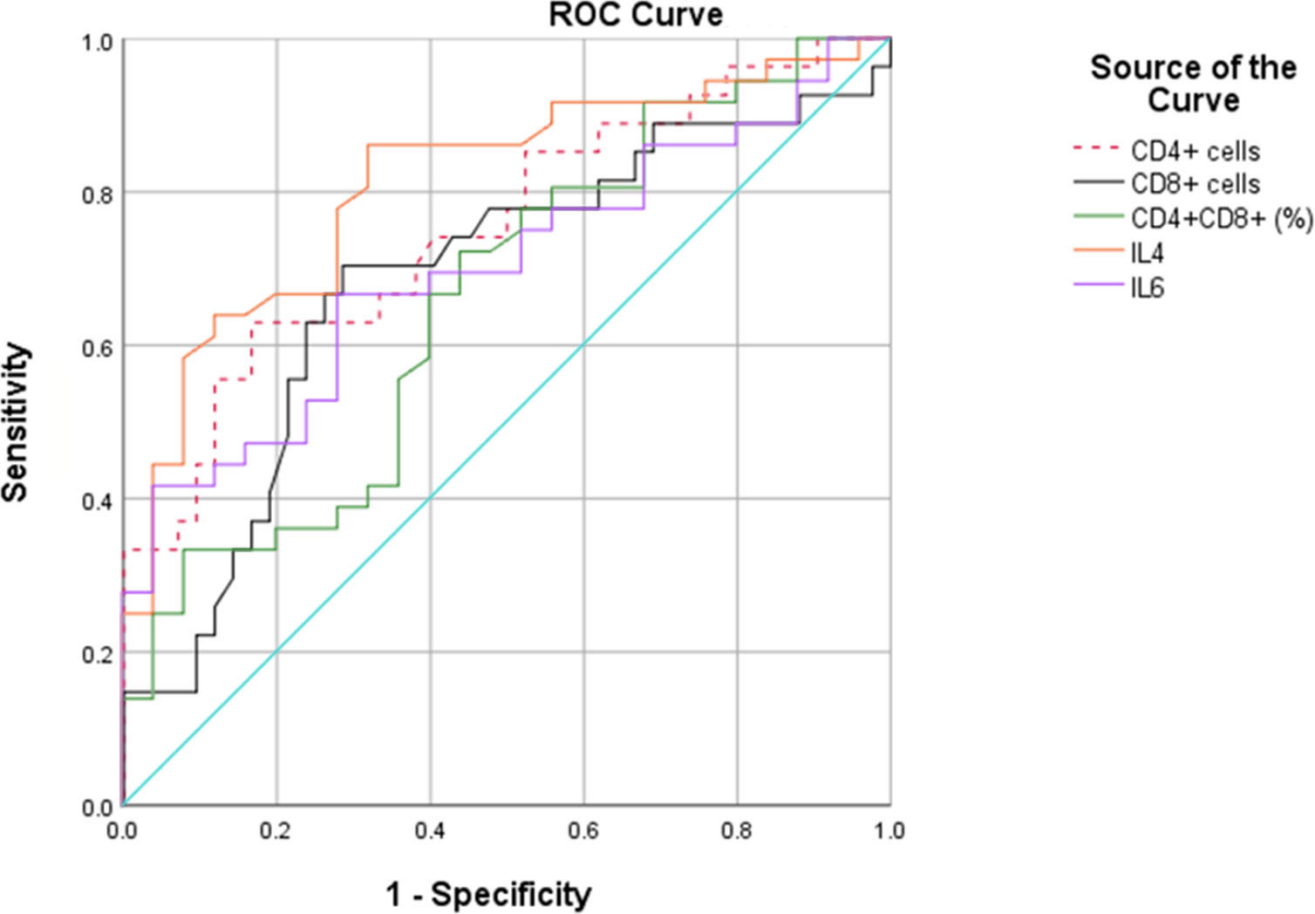
ROC curves for the performance of different parameters for coinfection.

We then performed ROC curves to determine the best cut-off for all parameters that were statistically significant between the two groups (**Figure 1**, **Table 2**). The IL4 level of 0.535pg/mL and a CD4+ cell counts of 199.5cells/µL provided the best overall accuracy. The AUC of the IL4 level was 81.6%, with a positive predictive value (PPV) of 81.4% and a negative predictive value (NPV) of 76.9%. The AUC of the CD4+ cell count was 75.4%, with a PPV of 74.5% and an NPV of 72%. The other thresholds were CD8+ cell counts < 124.5cells/µL, CD4+CD8+ (% lymphocytes) > 3.915 and IL6 > 388.9 pg/mL. The proportion of CD4+CD8+ cells had the highest specificity with a PPV of 92.3%; however, the NPV was only 50%.

**Table 2:** Accuracy and predictive values of ROC curve analysis.

Variable	Threshold	Sensitivity	Specificity	PPV	NPV	+LR	-LR	Youden index	AUC
CD4+ cells (cells/µL)	199.5	63%	83.3%	74.5%	72%	3.77	0.44	0.463	75.4%
CD8+ cells (cells/µL)	124.5	70.4%	71.4%	78.9%	62.5%	2.46	0.41	0.418	68.5%
CD4+CD8+ (% lymphocytes)	3.915	27.9%	96.9%	92.3%	50%	9	0.74	0.248	63.2%
IL4 (pg/mL)	0.535	85%	71.4%	81.4%	76.9%	2.97	0.21	0.564	81.6%
IL6 (pg/mL)	388.9	37.5%	96.4%	94.1%	56.9%	10.42	0.65	0.339	66.6%

PPV, positive predictive value; NPV, negative predictive value; +LR, positive likelihood ratio; -LR, negative likelihood ratio; AUC, areas under the ROC curve.

We then conducted a stepwise logistic regression analysis to identify independent associations between co-infection and the variables (**Table 3**). The results showed that CD4+ cell counts < 199.5cells/µL, CD8+ cell counts < 124.5cells/µL, IL4 > 0.535 pg/mL, IL6 > 388.9 pg/mL could be independent risk factors for coinfection.

**Table 3:** Stepwise, backward regression analysis with all possible confounders.

Dependent variable	OR (95% CI)	Standard error	*p* value
CD4+ cell counts < 199.5cells/µL	9.015 (1.2-67.734)	1.029	0.033
CD8+ cell counts < 124.5cells/µL	9.007 (1.181-68.683)	1.037	0.034
IL4 > 0.535 pg/mL	15.116 (1.927-118.555)	1.051	0.010
IL6 > 388.9 pg/mL	16.756 (1.018-275.682)	1.429	0.049

Within the coinfection group, only nine (16.7%) patients survived, while the other 45 (83.3%) patients died during hospitalization (**Table 4**). Age, sex, BMI, and the proportion of participants with comorbidities were not significantly different between the deceased and survived groups. However, we observed that deceased patients had lower levels of total lymphocytes, T cells, and albumin.

**Table 4:** Clinical characteristics and laboratory indicators in the survived and deceased groups .

Variable	Survived	Deceased	*P-value*
Patients(n)	9	45	
Demographics			
Age (year, $\bar{x}\;\pm \;s$ )	70.33 ± 15.04	73.42 ± 15.64	0.589
Male [n (%)]	8 (88.9%)	38 (84.4%)	1.000
Comorbidity			
Cancer [n (%)]	0 (0%)	2 (4.4%)	1.000
Diabetes mellitus [n (%)]	3 (33.3%)	14 (31.1%)	1.000
Hypertension	5 (55.6%)	25 (55.6%)	1.000
Chronic pulmonary disease	0 (0%)	11 (24.4%)	0.227
Chronic kidney disease	0 (0%)	7 (15.6%)	0.469
Laboratory parameters			
Leukocytes (*10^9^/L, $\bar{x}\;\pm \;s$ )	11.22 ± 7.85	11.2 ± 6.00	0.993
Neutrophils (*10^9^/L, $\bar{x}\;\pm \;s$ )	9.46 ± 7.07	10.03 ± 5.66	0.792
Lymphocytes (*10^9^/L, $\bar{x}\;\pm \;s$ )	1.20 ± 0.87	0.64 ± 0.47	0.008
Platelet (*10^9/L, $\bar{x}\;\pm \;s$ )	168.67 ± 84.83	171.64 ± 68.09	0.909
C-reactive protein (mmol/L, $\bar{x}\;\pm \;s$ )	82.37 ± 68.13	116.45 ± 74.85	0.214
Procalcitonin (ng/mL, $\bar{x}\;\pm \;s$ )	1.14 ± 2.56	11.69 ± 29.56	0.294
Creatinine (µmol/L, $\bar{x}\;\pm \;s$ )	82.77 ± 27.31	191.58 ± 246.04	0.194
ALB (g/L, $\bar{x}\;\pm \;s$ )	34.79 ± 8.03	29.80 ± 4.64	0.013
LDH (U/L, $\bar{x}\;\pm \;s$ )	421.89 ± 150.17	509.40 ± 252.22	0.321
BNP (pg/mL, $\bar{x}\;\pm \;s$ )	222.52 ± 306.25	413.30 ± 846.38	0.590
T lymphocytes (% lymphocytes, $\bar{x}\;\pm \;s$ )	64.50 ± 12.25	58.80 ± 17.09	0.353
CD4+ (% lymphocytes, $\bar{x}\;\pm \;s$ )	31.70 ± 14.02	33.39 ± 11.89	0.715
CD8+ (% lymphocytes, $\bar{x}\;\pm \;s$ )	32.33 ± 14.09	26.02 ± 12.08	0.182
CD4-CD8- (% lymphocytes, $\bar{x}\;\pm \;s$ )	3.42 ± 2.09	3.36 ± 4.88	0.970
CD4+CD8+ (% lymphocytes, $\bar{x}\;\pm \;s$ )	2.63 ± 2.07	3.94 ± 4.45	0.399
T lymphocytes in blood (cells/µL, $\bar{x}\;\pm \;s$ )	368.00 ± 206.84	235.28 ± 146.46	0.042
CD4+ in blood (cells/µL, $\bar{x}\;\pm \;s$ )	151.56 ± 93.19	112.91 ± 70.26	0.181
CD8+ in blood (cells/µL, $\bar{x}\;\pm \;s$ )	120.56 ± 78.71	90.88 ± 62.25	0.238
Ratio CD4+/CD8+ in blood [*M* (*Q1*, *Q3*)]	0.85 (0.64,1.19)	1.28 (0.92,1.87)	0.163
IL2 (pg/mL, $\bar{x}\;\pm \;s$ )	1.35 ± 0.61	1.47 ± 1.02	0.773
IL4 (pg/mL, $\bar{x}\;\pm \;s$ )	0.89 ± 0.29	1.00 ± 0.63	0.625
IL6 (pg/mL, $\bar{x}\;\pm \;s$ )	435.86 (9.61,777.72)	106.74 (60.45,450.74)	0.906
IL10 (pg/mL, $\bar{x}\;\pm \;s$ )	42.83 (5.95,112.88)	12.96 (4.52,135.85)	0.424
TNF-α (pg/mL, $\bar{x}\;\pm \;s$ )	0.01 (0.01,1.08)	0.48 (0.01,1.32)	0.445

## Discussion

The presence of bacterial coinfection has been proved to be associated with increased mortality. A previous study revealed an excess of 50% deceased COVID-19 patients compared with patients without coinfection (11). Our study showed an incidence of 55.1% among patients with a confirmed bacterial infection during admission. Consistent with previous studies (5), more patients in the coinfection group died during hospital admission, which is most likely due to cytokine dysregulation, changes to immune cell activation and function, mucociliary dysfunction, and alterations to the respiratory tract epithelium (12). Procalcitonin (PCT) is used as a biomarker to predict bacterial co-infection. However, the results showed that it is not reliable (13). Patients with severe COVID-19 may have elevated PCT levels, but this does not seem to correlate with the presence of bacterial infection. Elevated C-reactive protein (CRP) and ferritin levels have been found in patients with coinfection; however, elevation did not occur before the diagnosis of infection (4). Likewise, neither PCT nor CRP levels were considered to have the ability to predict the onset of infection in our study.

Adaptive immune responses are important in controlling viral infections that cause diseases in humans. Lymphocytopenia is considered to be a prominent cause of severe COVID-19 (14). T lymphocyte subsets are key components of the adaptive immune system and are important factors in killing infected cells and supporting antibody-generating B cells (15). T cell dysfunction was considered to be highly connected and associated with COVID-19 severity (10). Previous studies (1, 16) have found that CD4+ and CD8+ T cells are significantly decreased in severe patients compared to non-severe patients, and are independently predictive of patient outcomes. We found that patients with co-infection had significantly lower levels of lymphocytes and T cells. However, no study has examined the relationship between T cell subsets and coinfection in patients with COVID-19. In our study, we revealed that patients with lower levels of CD4+ T cells and CD8+ T cells were more prone to co-infection with bacteria. Given that the immunosuppression could hamper bacterial clearance by inhibiting neutrophil and T-cell responses, we suggested that CD4+T cell counts lower than 199.5cells/µL, CD8+ T cells < 124.5cells/µL could be strong predisposing factors for the coinfection. Moreover, using two-color fluorescence analysis, peripheral CD4+CD8+ double-positive (DP) T cells were first found in humans in 1986 (17), and their levels vary depending on tissue distribution, health status, and age (18). However, the function of CD4+CD8+ DP T cells has not yet been clearly described and remains controversial in different studies. Cytotoxicity (19, 20) and suppressive roles (21, 22) are the two most common views. In our study, patients with co-infections tended to have a higher proportion of CD4+CD8+ DP T cells. We also found higher level of IL4 in patients with co-infections. This was consistent with a previous study that revealed that CD4+CD8+ DP T cells can produce elevated levels of IL-4, but not IFN-γ, IL-2, or IL-10, compared with CD4+ T cells or CD8+ T cells (23). Therefore, our results support a cytotoxic role for CD4+CD8+ DP T cells which expanded in response to SARS-CoV-2.

The levels of IL-1β, IL-2R, IL-6, and TNF-α were found to be significantly higher than the upper limits of normal in patients with confirmed secondary infections (24, 25). In another study, increased IL-6, IL-8, IL-10, and IFN-γ levels were found in severe patients compared to those in non-severe patients (1). We showed that patients with coinfection had higher level of IL4 and IL6 on the day of admission. IL-6 plays an important role in initiating antibacterial inflammation and can trigger a cascade of inflammatory mediators (26), is negatively correlated with NK cells and CD8+ T cells (27) and is considered to have the ability to predict the development of fatal SARS-CoV-2 pneumonia (28). Therefore, clinicians expected that interleukin-6 receptor blockade (Tocilizumab) could interrupt this inflammatory cascade at a early stage. However, even though several retrospective observational studies showed a positive effect on mortality, one randomised double-blind, placebo-controlled study suggested they found no significant effect on 28-day survival in severe COVID-19 cases while another two clinical trials were stopped early after an increased number of deaths in the tocilizumab group (29). Another prospective clinical trial in moderately ill patients hospitalized with COVID-19 also suggested that tocilizumab was not effective for preventing intubation or death (30). Meanwhile, IL-4 exerts both immunostimulatory and immunosuppressive activities. Previous studies have suggested that IL‐4 shown a protective role in acute lung injury, acute kidney injury and influenza/S. pneumoniae co‐infection (31). In our study, IL4 andIL6 were found to be potential biomarkers for predicting the development of coinfection in patients with COVID-19. And IL6 > 388.9 pg/mL and IL4>0.535pg/mL were independent risk factors for co-infection.

Our study had several limitations. First, it included a small number of patients. These results should be verified in larger cohorts. Second, our study lacks follow-up data. We did not follow the patient survival after discharge. Third, CD4+ and CD8+ lymphocyte subpopulations were not detected. Further mechanistic studies are needed to determine the exact role of the T cell subsets. Therefore, a larger patient cohort with more laboratory tests is warranted to validate our findings.

## Conclusion

In conclusion, the levels of lymphocyte subsets and cytokines can be used as biomarkers for predicting bacterial coinfection in patients with critical COVID-19. Patients with lower levels of total lymphocytes and T cells tend to have poor prognosis. Therefore, monitoring the levels of immune inflammatory factors might be important for assessing the development and prognosis of coinfection in patients with critical COVID-19.

## Data Availability

The raw data supporting the conclusions of this article will be made available by the authors, without undue reservation.
